# Effect of Complexes of Cobalt With Aminoacids on the Replication of Herpes Simplex Virus Type 1 (HSV-1)

**DOI:** 10.1155/MBD.1996.149

**Published:** 1996

**Authors:** T. Varadinova, S. Shishkov, M. Panteva, P. Bontchev

**Affiliations:** 1 Laboratory of Virology Faculty of Biology Sofia University 8 Dragan Tzankov Blvd 1 Sofia 1421 Bulgaria; 2 Faculty of Chemistry Sofia University 8 Dragan Tzankov Blvd 1 Sofia 1421 Bulgaria

## Abstract

Cobalt, being essential metal, influences different physiological and enzymatic functions. As cobalt does not accumulate in the body, Co-compounds have relatively low toxicity. The aim of the present study is the effect of complexes of Co(II) with aminoacids - lysine, arginine, histidine and serine on HSV-1 replication. No effect of [O_2_Co(his)_4_].nH_2_O and [O_2_Co(arg)_2_].nH_2_O on HSV-1 infection *in vitro* was found. Both, [O_2_Co(lys)_2_].nH_2_O and [O_2_Co(ser)_2_].nH_2_O suppress the attachement of HSV-1 particles onto target cells and the viral replication as well. Moreover, the properties of the particular Co-complex (charge, stability, structure) are manifestated by their virucidal effect. Thus, [O_2_Co(ser)_2_].nH_2_O irreversibly inhibits the infectious activity of free HSV-1 virions, while virucidal effect of [O_2_Co(lys)_2_].nH_2_O is completely reversible after the 2h of contact.


### EFFECT OF COMPLEXES OF COBALT WITH AMINOACIDS ON THE REPLICATION OF HERPES SIMPLEX VIRUS TYPE 1 (HSV-1)

T. Varadinova*[1], S. Shishkov[1], M. Panteva[1], and P. Bontchev[2]

[1] Laboratory of Virology, Faculty of Biology, Sofia University; [2] Faculty of Chemistry , Sofia University
8 Dragan Tzankov Blvd 1 , 1421 Sofia , Bulgaria

### Abstract

Cobalt, being essential metal, influences different physiological and enzymatic functions. As cobalt does not accumulate in the body, Co-compounds have relatively low toxicity. The aim of the present study is the effect of complexes of Co(II) with aminoacids - lysine, arginine, histidine and serine on HSV-1 replication. No effect of $[O_2Co(his)_4].nH_2O$ and $[O_2Co(arg)_2].nH_2O$ on HSV-1 infection *in vitro* was found. Both, $[O_2Co(lys)_2].nH_2O$ and $[O_2Co(ser)_2].nH_2O$ suppress the attachement of HSV-1 particles onto target cells and the viral replication as well. Moreover, the properties of the particular Co-complex (charge, stability, structure) are manifestated by their virucidal effect. Thus, $[O_2Co(ser)_2].nH_2O$ irreversibly inhibits the infectious activity of free HSV-1 virions, while virucidal effect of $[O_2Co(lys)_2].nH_2O$ is completely reversible after the 2h of contact.

### Introduction

Apart from the importance of cobalt for functional activity of vitamin $B_{12}$ (1) this metal ion influences different physiological and enzymatic functions. Thus, cobalt speeds up ATP turnover (2), activates arginase and inhibits δ-aminolevulinic acid synthase (3), affects mixed-function oxidase of the liver (4), enhances acylamino acid hydrolase (5) and yeast enolase (6). Cobalt expresses its biological effects through the cytoplasm of the cell (7). Because cobalt does not accumulate in the body, acute fatal intoxications have not yet been reported. That is why cobalt compounds have relatively low toxicity (8).

Up to now, there are no data on the effect of cobalt complexes and compounds on Herpes simplex virus (HSV) infection. Aminoacids, being common ligands for essential metal ions and for Co(II) in particular (7), are responsible for a proper function of the ion. That is why we decided to study the *in vitro* effect of complexes of Co(II) with aminoacids lysine, arginine, histidine and serine on HSV-1 infection.

### Materials and methods

**Virus and cells.** HSV-1 (strain Victoria), primary rabbit kidney cells (r.k.) and diploid strain from human embrional lung fibroblasts (F) were used.

**Complexes of Co(II) .** The following Co-complexes were specially synthesized: with essential aminoacids lysine, arginine, histidine and with replaceable aminoacid serine.

**Maximal nontoxic concentration** (MNC). Tube cells from suspension were influenced with an appropriate Co-complex in the following effective concentrations: 1000, 100, 10, 1 and 0.1μM. Cells were cultured at 37°C. The degeneration of monolayer and changes in cell morphology were examined microscopically from the 24th till the 96th hour. Each experiment was duplicated. The highest concentration in the presence of which neither changes in cell morphology nor degeneration of monolayer was found during the whole period of investigation as compared to the control - cells growing in nonmodified medium, is recognized as MNC.

**Infectious virus titre** was determined on the 48 h after the infection and expressed in $\log_{10}$ pfu/0.1ml .

**Effect of Co-complexes on the replication of HSV-1**. Cells from suspension were influenced with virus stock in ten-fold dilutions and appropriate Co-complexes in nontoxic concentrations. The effect on viral replication was determined on the 48h after culturing at 37°C by reduction of infectious virus titre as compared to the viral control - virus-infected untreated cells.

**Reversibility of the inhibitory effect.** After studying the direct effect of Co-complexes on the replication of HSV-1, the samples containing 100 pfu/0.1ml of HSV-1 and appropriate concentrations of $[O_2Co(lys)_2].nH_2O$ and $[O_2Co(ser)_2].nH_2O$, as well as that from untreated viral controls were frozen and thawed. Cells F suspended in nonmodified medium were infected with ten-fold dilutions of each sample. The reversibility of the effect on the replication of HSV-1 was determined by infectious virus titres in the experimental samples as compared to that from the untreated control.

**Effect on the adsorption.** Tube F cells were influenced with 100 pfu/0.1ml HSV-1 and 10μM of $[O_2Co(lys)_2].nH_2O$ or $[O_2Co(ser)_2].nH_2O$. At the 15, 30, 45, 60 and 120min after culturing at room temperature cells were washed with Haenks solution, covered with nonmodified medium and cultured for 48h at 37°C. Samples were frozen and thawed. Cells from suspension were infected with ten-fold dilutions of each sample. Infectious virus titres were determined at the 48h and compared to that from viral control - infected cells cultured in nonmodified medium at the above intervals.

**Effect of Co-complexes on extracellular (free) HSV-1 virions (virucidal effect).** Equal volumes of HSV-1 stock containing 100 pfu/0.1ml and media modified with 10μM of $[O_2Co(ser)_2].nH_2O$ or $[O_2Co(lys)_2].nH_2O$ were incubated at 37°C for 15, 30, 60 and 120min. Cells from suspension were infected with ten-fold dilutions of each sample and cultured for 48 h at 37°C. The virucidal effect was determined by the reduction of infectious virus titres as compared to that of the viral control - equal volumes of HSV-1 stock and nonmodified medium incubated as described above.

### Results

MNC of complexes of Co(II) with essential aminoacids lysine, arginine and histidine is 10μM. The complex of Co(II) with replaceable aminoacid serine is 10 times more toxic than that of the first ones (MNC = 1μM). Neither cell-determined susceptibility nor resistance to the complexes studied was found (data not shown).

Table 1
Effect of complexes of cobalt with amino acids lysine, arginine, histidine and serine on the replication of HSV-1*

| Complex | Concentration, μM | Infectious virus titre, $log_{10}$pfu/0.1ml | Inhibition, % |
|---|---|---|---|
| $[O_2Co(lys)_2].nH_2O$ | 10 | 4.9 | 99 |
| | 1 | 5.8 | 90 |
| | 0.1 | 6.6 | 50 |
| | 0.01 | 6.7 | 20 |
| $[O_2Co(ser)_2].nH_2O$ | 1 | 6.0 | 90 |
| | 0.1 | 6.8 | 0 |
| $[O_2Co(arg)_2].nH_2O$ | 10 | 6.9 | 0 |
| $[O_2Co(his)_4].nH_2O$ | 10 | 6.9 | 0 |
| HSV-1 control | | 6.9 | |

* - data from the experiments done on F cells. Equal results are found on r.k. cells.

Complexes of Co(II) with arginine and histidine have no effect on the replication of HSV-1 during multicycle growth (tables 1 and 2). The complex of Co(II) with lysine inhibits HSV-1 replication in dose-dependent manner. The inhibitory effect is independent on the infectious dose (6.9 or 4.7 $log_{10}$ pfu/0.1ml) and is irreversible when the complex is applied in MNC.

Table 2
Reversibility of the anti-HSV-1 effect of $[O_2Co(lys)_2].nH_2O$ and $[O_2Co(ser)_2].nH_2O$*

| Complex, μM | Effect on the replication | | Reversibilityof the action | |
|---|---|---|---|---|
| $[O_2Co(lys)_2].nH_2O$ | | | | |
| 10 | 2.3[a] | 99.6[b] | 1.6[a] | 90[b] |
| 1 | 2.8 | 99 | 2.9 | 21 |
| 0.1 | 3.0 | 98 | 2.9 | 21 |
| 0.01 | 4.5 | 27 | 3.3 | 0 |
| $[O_2Co(ser)_2].nH_2O$ | | | | |
| 1 | 2.5 | 99.3 | 1.9 | 90 |
| 0.1 | 2.9 | 98 | 2.8 | 27 |
| 0.01 | 3.3 | 96 | 3.0 | 0 |
| 0.001 | 4.3 | 60 | n.d | |
| HSV-1 control | 4.7 | | 3.0 | |

* data from the experiments done on F cells
[a] infectious titre in $log_{10}$ pfu/0.1ml ; [b] inhibition; %; n.d.: not done

In contrast, the inhibitory effect of $[O_2Co(ser)_2].nH_2O$ strongly depends on the infectious virus dose. Thus, when cells were treated with the complex and 4.7 $log_{10}$ pfu/0.1ml HSV-1 the degree of the inhibition of virus replication is dose-dependent. Furthermore, when this complex is applied to cells infected with 6.9 $log_{10}$ pfu/0.1ml the inhibition of virus replication is obtained under the action of MNC (1μM) only. This concentration irreversibly affects virus growth as well. As complexes of Co(II) with arginine and histidine had no effect on the replication of HSV-1, the influence of these complexes on the adsorption of virus particles onto host cells, as well as on the infectivity of the extracellular virions was not studied.
As is shown in table 3 and fig.1 $[O_2Co(lys)_2].nH_2O$ and $[O_2Co(ser)_2].nH_2O$ suppress the process of irreversible adsorption of HSV-1 particles onto host cells. Thus, at the end of the adsorption period - 1h, up to 10% viral particles were steadily attached. The data on the quantity of adsorbed viral particles after the 2h show that the suppressive effect of the above complexes is irreversible.

Table 3

Effect of $[O_2Co(lys)_2].nH_2O$ and $[O_2Co(ser)_2].nH_2O$ on the adsorption of HSV-1*

| Complex | Duration of the adsorption, min | | | | |
|---|---|---|---|---|---|
| | 15 | 30 | 45 | 60 | 120 |
| $[O_2Co(lys)_2].nH_2O$, 10μM | 1.8** | 1.9 | 2.5 | 2.3 | 2.3 |
| $[O_2Co(ser)_2].nH_2O$, 10μM | 1.6 | 2.0 | 2.0 | 2.0 | 2.3 |
| HSV-1 control | 1.8 | 2.5 | 2.8 | 3.3 | 3.3 |

* - data from experiments done on F cells.
** - infectious titre in $log_{10}$ pfu/0.1ml

Virucidal effect of complexes of Co(II) with lysine and serine is shown in table 4 and fig.2. Soon after the contact (15 - 30 min) 60-75% of HSV-1 virions lost the ability to infect cells. With prolongation of the contact the virucidal effect of $[O_2Co(lys)_2].nH_2O$ progressively decreases and after120min no inactivation of free HSV-1 virions was obtained. Conversely, the virucidal effect of $[O_2Co(ser)_2].nH_2O$ is expressed during the whole period of investigation when up to 60% of the virions were unable to infect cells.

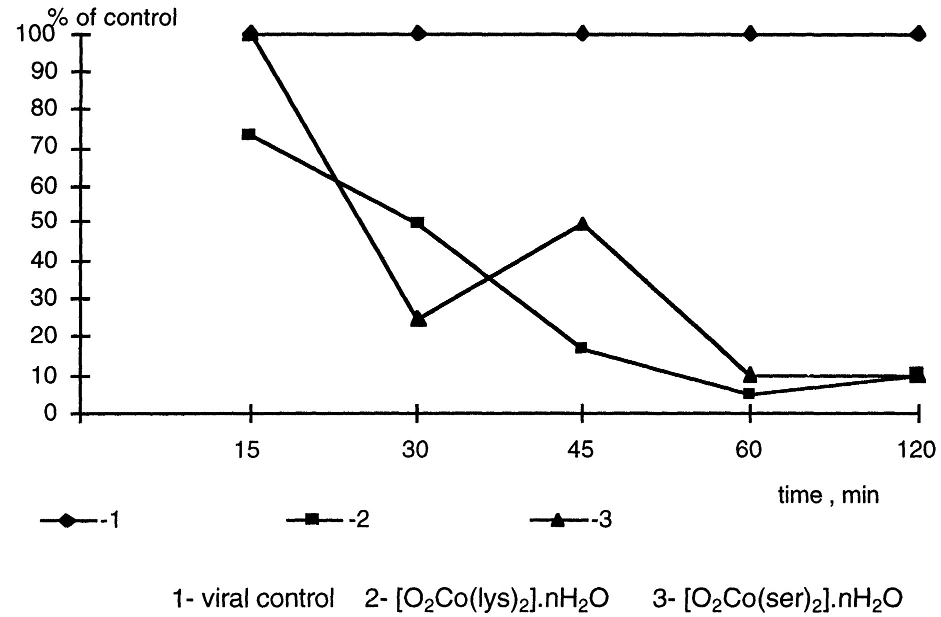

Fig.1 Effect of $[O_2Co(lys)_2].nH_2O$ and $[O_2Co(ser)_2].nH_2O$ on the attachement of HSV-1

Table 4
Effect of $[O_2Co(lys)_2].nH_2O$ and $[O_2Co(ser)_2].nH_2O$ on free HSV-1 virions *

| Complex | Duration of the contact, min | | | |
|---|---|---|---|---|
| | 15 | 30 | 60 | 120 |
| $[O_2Co(lys)_2].nH_2O$, 10 μM | 4.5** | 3.6 | 3.0 | 2.9 |
| $[O_2Co(ser)_2].nH_2O$, 10 μM | 4.5 | 3.6 | 3.2 | 2.5 |
| HSV-1 control | 5.1 | 4.0 | 3.5 | 2.9 |

* data from experiments done on F cells.
** infectious titre in $\log_{10}$ pfu/0.1ml

### Discussion

The specific biological effect of the different complexes of cobalt is a result of the combination of the metal and the ligand in a new species - the complex, which (depending on its properties, such as charge, stability and structure) could express a particular activity. The following set of results is in accordance with this suggestion.

1. Complexes of Co(II) with essential amino acids lysine, arginine and histidine are less cytotoxic (MNC 10μM) than the complex with serine (MNC 1μM). The highest toxicity of $[O_2Co(ser)_2].nH_2O$ for r.k. and F cells is probably due to the specificity of the ligand - a replaceable, small amino acid -serine.

2. Complexes of Co(II) with arginine and histidine had no effect on HSV-1 infection *in vitro*. In contrast, $[O_2Co(lys)_2].nH_2O$ and $[O_2Co(ser)_2].nH_2O$ strongly and irreversibly inhibited HSV-1 replication when applied in MNC - 10μM and 1μM respectively. In addition, the inhibitory effect of $[O_2Co(ser)_2].nH_2O$ depends on the infectious virus dose, while that of $[O_2Co(lys)_2].nH_2O$ is dose-independent.

3. In order to study the effect of the above complexes on extracellular virions, as well as on the attachment of viral particles onto target cells a different set of experiments was done. The results show that $[O_2Co(lys)_2].nH_2O$ and $[O_2Co(ser)_2].nH_2O$ suppress the attachment of HSV-1 particles onto target cells in the same degree. On the other hand, these two complexes express different effect on extracellular virions. Thus, $[O_2Co(ser)_2].nH_2O$ inactivates infectious activity of up to 50% of HSV-1 virions after 30, 60 and 120min prolonged contact. Furthermore, the virucidal effect of $[O_2Co(lys)_2].nH_2O$ is completely reversible after the 2h prolonged contact.
Put together, the data show that $[O_2Co(lys)_2].nH_2O$ affects HSV-1 infection through the plasma membrane and/or cytoplasm. At the level of plasma membrane the complex suppresses the adsorption of HSV-1 particles, thus sharply reducing the quantity of viruses entering the target cell.

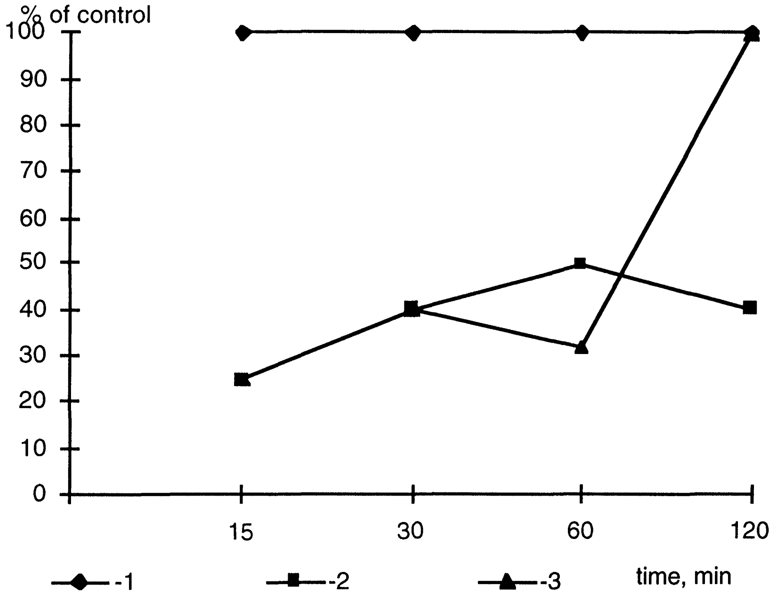

1-viral control 2-$[O_2Co(ser)_2].nH_2O$ 3-$[O_2Co(lys)_2].nH_2O$

Fig.2 Virucidal effect of $[O_2Co(ser)_2].nH_2O$ and $[O_2Co(lys)_2].nH_2O$

Furthermore, the ligand of Co(II) - lysine, could ensure the penetration of $[O_2Co(lys)_2].nH_2O$ through plasma membrane. Localised inside the cell (probably in the cytoplasm - a natural site of cobalt) this complex could be responsible for further inhibition on virus replication. This suggestion is confirmed by the fact that the anti-HSV-1 effect is independent of the infectious dose.
Conversely, the inhibition of the infectivity of the free virions, as well as of the attachement of HSV-1 particles onto target cells show that the other complex, $[O_2Co(ser)_2].nH_2O$, affects HSV-1 infection through membranes - cellular and viral. Moreover the ligands serine and lysine, but not histidine and arginine ensure anti-HSV-1 effect of the appropriate Co-complex through different target sites. Anti-HSV-1 activity of these complexes is independent on the specificity of the cells used.

### References

1. J. Angerer and R. Heinrich, in " *Handbook on Toxicity of Inorganic Compounds*", **20**, 251, (1988)
2. G. R. Meyer and R. Cornelius, *J. Inorg. Biochem.*, **22**, 249 (1984)
3. N. Schoenfeld, Y. Greenblat, O. Epstein, D. P.Tschudy and A. Atsmon, *Biochem. Pharmacol.*, **32**, 2333 (1983)
4. D. A. Schoeller, A. N. Kotake, G. H. Lambert, P. S. Krager and A. L. Baker, *Hepatology*, **5**, 276 (1985)
5. J. Gilles, H. G. Loffler and F. Schneider, *Z. Naturforsch.*, **C 39**, 1017 (1984)
6. S. L. Rose, L. Ch. Dickinson and E. W. Westhead, *J. Biol. Chem.*, **259**, 4405 (1984)
7. *"The Biological Chemistry of the Elements. The Inorganic Chemistry of Life"*, J. J. R. F. Da Silva, R. J. P. Williams, Eds., 23, Claredon Press, Oxford, (1993)

8. " *NIOSH Registry of Toxic Effects of Chemical Substances",* Washington, D. C. (1976)

**Received: February 29, 1996 - Accepted: March 22, 1996 - Received in revised camera-ready format: May 18, 1996**

